# The Effect of Early Maternal Separation Combined With Adolescent Chronic Unpredictable Mild Stress on Behavior and Synaptic Plasticity in Adult Female Rats

**DOI:** 10.3389/fpsyt.2021.539299

**Published:** 2021-03-05

**Authors:** Jiawen Huang, Chongkun Shen, Ran Ye, Yafei Shi, Weirong Li

**Affiliations:** ^1^Science and Technology Innovation Center, Guangzhou University of Chinese Medicine, Guangzhou, China; ^2^Institute of Clinical Pharmacology, Guangzhou University of Chinese Medicine, Guangzhou, China; ^3^School of Fundamental Medical Science, Guangzhou University of Chinese Medicine, Guangzhou, China; ^4^Heyuan People's Hospital, Heyuan, China; ^5^School of Pharmaceutical Sciences, Guangzhou University of Chinese Medicine, Guangzhou, China

**Keywords:** maternal separation, depression, anxiety, synaptic plasticity, early stress

## Abstract

Our aims were to evaluate the depression model of early maternal separation (MS) combined with adolescent chronic unpredictable mild stress (CUMS) in female adult SD rats to observe the behavior and the expressions of synaptic proteins in rats and to provide a reference for the screening of antidepressant drug activity. In our study, MS and CUMS were conducted to establish a dual stress model on female rats. Behavioral tests, including the sucrose preference test, open field test, and zero maze test, were used to detect depression-like and anxiety-like behavior of animals. Nissl staining was used to detect the number of neuronal cells in the hippocampus CA1 and DG regions of rats from each group. Synaptophysin (SYN), postsynaptic density-95 (PSD-95), and growth-associated protein-43 (GAP-43) expressions in the hippocampus were detected by western blot. Expression of the hippocampus SYN protein was further detected by immunohistochemistry. Rats in the MS+CUMS group presented more serious depression-like and anxiety-like behavior than in the MS group. Also, few Nissl bodies in the hippocampus CA1 and DG regions, less percentage of SYN-positive cells, and downregulated expressions of SYN, PSD-95, and GAP43 were found in the hippocampus of rats in MS+CUMS group. In conclusion, adult female rats that underwent MS and CUMS performed more critical depression-like and anxiety-like behaviors, and this process may be resulted from synaptic plasticity impairment.

## Introduction

Depression is a common psychiatric disorder with a high incidence, and its pathogenesis is still not clear. The World Health Organization points out that depression would be one of the three major diseases in 2030 (1). However, due to the complexity of the etiology of depression, how to establish an effective model of depression has become one of the key issues when researching depression. Additionally, although the etiology of depression has not been confirmed, most scholars believe that the occurrence of depression is influenced by environment and heredity (2). Berton et al. found that genetic factors account for 40–50% of all factors leading to depression, and the remaining 50–60% are closely related to life stress in early childhood (3). Another study has demonstrated that the one who has adverse experience at a young age may have a risk of depression 4-fold higher than normal persons (4).

Contemporary studies on depression are mostly in male animals, while female animals are often excluded because of interference of estrogen (5–7). In fact, there are reports that women are twice as likely as men to experience depression and anxiety disorders (8, 9). So, it is more valuable to study depression in female rats. In addition, adolescence is the most critical period of physiological and psychological changes, and it plays an indispensable role in neurodevelopment and mental diseases. Maternal separation (MS) is a classical model of early stress study. Moreover, it is known that chronic unpredictable mild stress (CUMS) overcomes the stress habit and is commonly used to simulate depressive behavior (10). We chose female rats with MS combined with CUMS as experimental subjects of early stress. On the one hand, the complexity of the etiology of depression was in our consideration. On the other hand, it could also provide a novel model design for the construction of a new model to explore the impact of early stress on adult depression. As anxiety and depression often occur together (11), we would explore both depression-like and anxiety-like behaviors in our study.

Clinical studies have found that the hippocampus of depressed patients is reduced in volume (12, 13). Animal studies have found that neurons and glial atrophy are lost in the hippocampus of depression-like model animals (14). The reason may be that stress not only reduced dendritic spine density and number of branches but also thinned postsynaptic density (15). The hippocampus, a brain structure regulating stress and related to depression (16), is more vulnerable to stimulation damage (17), which may lead to neuron reduction (18, 19). Previous studies have shown that depression is closely related to the hippocampus synaptic plasticity (20, 21). Menard et al. have shown that the number of synapses in depression animals is significantly reduced, and the residual synaptic structure and function have different degrees of damage (22). Increasing evidence had found that dysregulation of synaptic plasticity is related to depression (21). However, there are so far only a few papers on synaptic plasticity proteins (2, 23). Thus, changes in synaptic plasticity proteins in the depression model are the focus of this study. This study provides a basis for research on synaptic plasticity protein-related depression and suggests that drugs may be able to improve depression by changing synaptic plasticity.

Based on the researches above, the stress model of MS, CUMS, and MS combined with CUMS were used in this study. We tried to observe the effects of different stress on the depression-like and anxiety-like behaviors of adult female rats and synaptic plasticity of the hippocampus.

## Materials and Methods

### Animals and Groups

**Figure 1:**
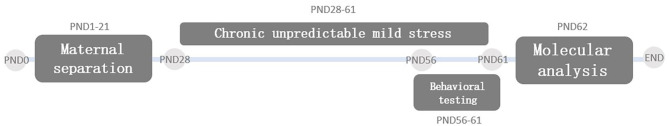
Study design flowchart. PND, postnatal day.

A total of 10 Sprague Dawley (SD) pregnant rats were purchased from the Experimental Animal Center of Guangzhou University of Chinese Medicine (China). Animals were housed in a constant animal facility at a temperature of 20–25°C, relative humidity of 50–60%, food and water *ad-libitum*, and a 12 h light/dark cycle. On the day the pups were delivered (PND0), only female pups were selected and then were divided into four groups (*n* = 8 animals/group) randomly: the non-maternal separation (CON group), maternal separation only group (MS group), chronic unpredictable mild stress only group (CUMS group), and maternal separation group plus chronic unpredictable mild stress (MS + CUMS group). The body weight was detected once a week. The study design flowchart is illustrated in Figure 1. After behavioral tests, on PND62, animals were anesthesia with 10% chloral hydrate, and the brain tissues were removed. All experimental protocols were approved by the Animal Experimental Committee of Guangzhou University of Chinese Medicine (Ethical approval number: 20190605015).

### Maternal Separation (MS)

The protocol of MS we used was established in our previous study (24). In brief, from PND1 to PND21, the pups in the MS group and MS+CUMS group were separated from their mother for a total of 360 min each day (8:00–11:00 a.m. and 14:00–17:00 p.m.). During separation time, these pups were placed into cages filled with cotton for the purpose of maintaining the temperature. Rats in the CON group and CUMS group remained with their mother and did not undergo any interference during the MS period. Since PND21, rats were put into cages without their mother with four pups per cage.

### Chronic Unpredictable Mild Stress (CUMS)

Similar to the previous studies (25, 26), from PND28, pups received one of nine trials (Table 1) every day until the end of the whole study (PND61). Each trial was randomly taken every day, and the same trial was not used twice in a row.

**Table 1:** Method for CUMS comprises nine trials.

**Trials**
1	Food deprivation for 24 h
2	Water deprivation for 24 h
3	Thermal water stimulation (45°C) for 5 min
4	Day and night reversal for 24 h
5	Ice water stimulation (4°C) for 5 min
6	Cage tilting 45° for 24 h
7	Wet padding for 24 h
8	Crowded squirrel cage for 24 h
9	Empty water bottle for 2 h

### Sucrose Preference Test (SPT)

On PND56, rats were given two bottles of 1% sucrose solution for 24 h; one bottle was replaced with pure water on PND57, and the position of two bottles was exchanged after 12 h; on PND58, food and water were deprived from rats for 24 h; on PND59, the rats were given a bottle of pure water and a bottle of 1% sucrose solution. After 2 h, the lost weight of each bottle was recorded to determine the intake of the rats. Sucrose Preference rate (%) = Sucrose consumption/(Sucrose consumption + water consumption) ^*^ 100%.

### Open Field Test (OFT)

OFT was conducted on PND56. The rats were placed in the center of a 100 × 100 × 60 cm black box, and the movement of each rat in the box was automatically recorded by the video-tracking analysis system for 3 min. The time in the central area and the total distance would be used as indicators.

### Zero Maze Test (ZMT)

ZMT was conducted on PND60. The maze (outer diameter: 100 × 100 cm; inner diameter: 80 × 80 cm) was divided into four quadrants (two are opposing open quadrants and two are opposing closed quadrants). Rats were placed in an open quadrant, and the tracking system was triggered (Shanghai Xinsoft Information Technology Co., Ltd., China). Each rat was tested for 5 min, and the time rat spent in open quadrants would be used as an index.

### Nissl Staining

On PND62, brain tissues were removed and then embedded and cut at 5 μm thickness. The staining was carried out with Nissl staining solution (Shanghai Biyuntian Biotechnology Co., Ltd., China) for 40 min at 37°C after the sections dewaxing and rehydration. The number of Nissl bodies in the CA1 and DG region was observed under a field of view of a microscope (Leica Microsystems Wetzlar GmbH) at 400 × magnification. Select cells that clearly observe the nucleus and nucleolus for statistics.

### Immunohistochemistry

The embedded brain tissues were cut into 5 μm sections. Firstly, after dewaxing, the sections were immersed in 0.01 M sodium citrate buffer solution at 95–100°C for 15 min and then incubated with 3% hydrogen peroxide treatment for 10 min. Secondly, after being incubated with goat serum working solution for 15 min, the sections were incubated with anti-rabbit SYN antibody (Affinity, USA) at 4°C overnight. Thirdly, on the following day, the sections were incubated with biotinylated IgG, streptavidin, and DAB following the DAB detection kit (ZSGB-BIO, China). The sections were digitized and analyzed by a Leica microscope (Leica Microsystems Wetzlar GmbH). Each section was imaged at 400 × magnification and measured the percentage of SYN-positive area in each field of view by quantitative image analysis with ImageJ 1.45 software.

### Western Blot

Total protein of the hippocampus was lysed by using RIPA lysis buffer, and protein concentrations were quantified by using a BCA kit (Beijing Dingguo Changsheng Biotechnology co., Ltd., China). Proteins were separated by sodium dodecyl sulfate polyacrylamide gel electrophoresis (SDS-PAGE), then transferred to PVDF membranes and blocked with 5% skim milk powder. Antibodies against SYN (Affinity (USA), AF0257-50; 1:1000), PSD-95 (Affinity (USA), AF5283-50; 1:1000), GAP-43 (Affinity (USA), DF7766-50; 1:1000), and Tubulin (Affinity (USA), AF7018-50; 1: 5000) were incubated with membranes overnight at 4°C. Finally, the IgG-HRP antibody (CST (USA), BST11L22C51, 1:5000) was incubated with membranes for 1 h at room temperature. After the above steps were completed, membranes were combined with ECL Plus reagent (Beijing Lanjieke Technology Co., Ltd.) for a color reaction. Besides, the gray value was quantified by using Image Lab software.

### Statistical Analysis

SPSS22 software was used to perform analysis and all data are expressed as mean ± standard error (SEM). One-way analysis of variance (ANOVA) was used for statistical analysis. When the variances were homogeneous, we used an LSD *post-hoc* test; otherwise, we used Dunnett's T3 test. The results were performed using GraphPad Prism 6.0. *p* < 0.05 was considered statistically significant.

## Results

### The Effect of MS and/or CUMS on Body Weight

**Figure 2:**
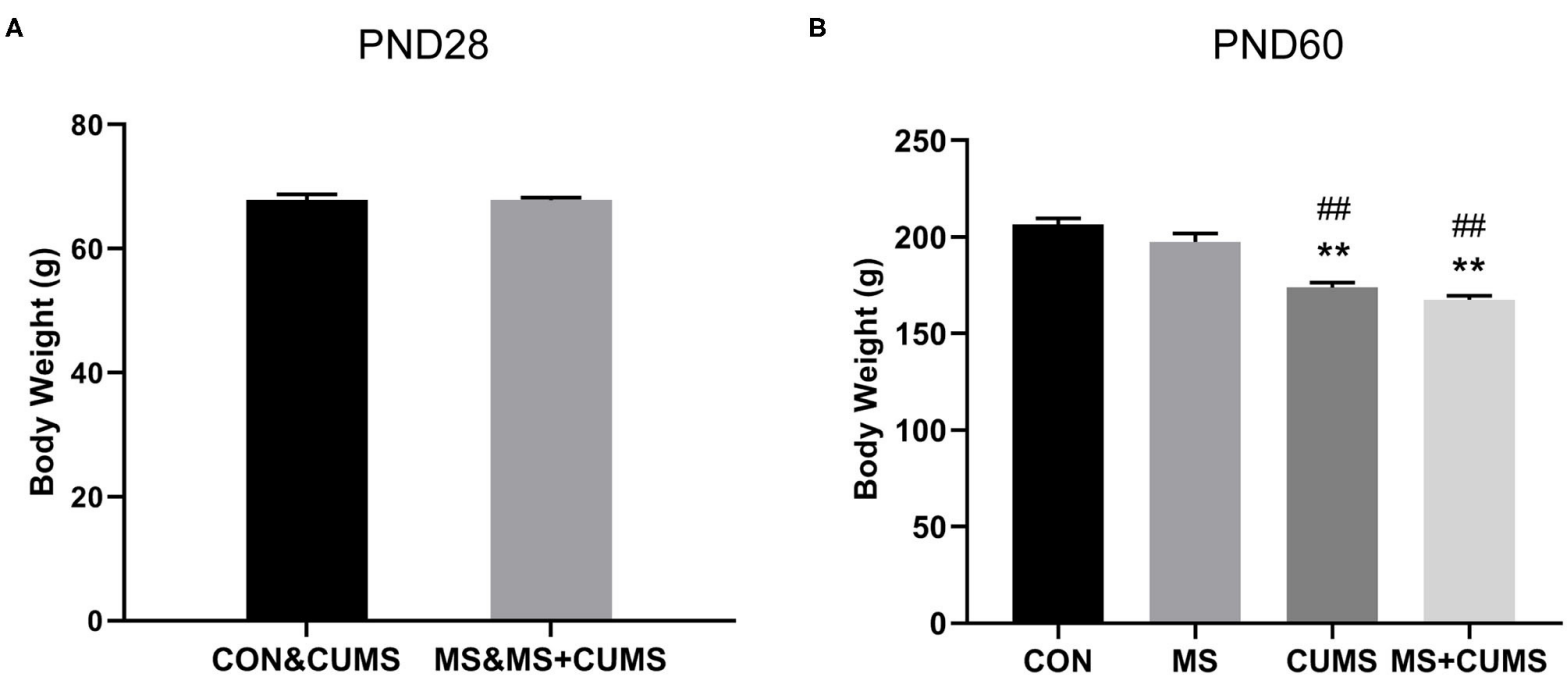
Body weight record of rats on PND28 **(A)** (*n* = 16) and PND60 **(B)** (*n* = 8). The values represent the mean ± SEM. ^**^p < 0.01 vs. CON; ^##^*p* < 0.01 vs. MS. CON, control; MS, maternal separation; CUMS, chronic unpredictable mild stress.

We observed the effect of MS and CUMS on body weight in this study, including PND28 and PND60 time points (Figure 2). As shown in Figure 2A, after MS for 28 days, rats in the MS group showed no significant influence on body weight compared with the non-MS group. After CUMS, rats in CUMS group [*F*_(3, 31)_ = 34.76, *p* < 0.01] and MS+CUMS group [*F*_(3, 31)_ = 34.76, *p* < 0.01] showed lower body weight than rats in the control group, while rats in the MS group showed no significant difference (Figure 2B). Moreover, there was no significant difference between CUMS and MS+CUMS groups on PND60.

### The Effect of MS and/or CUMS on Behavioral Tests

**Figure 3:**
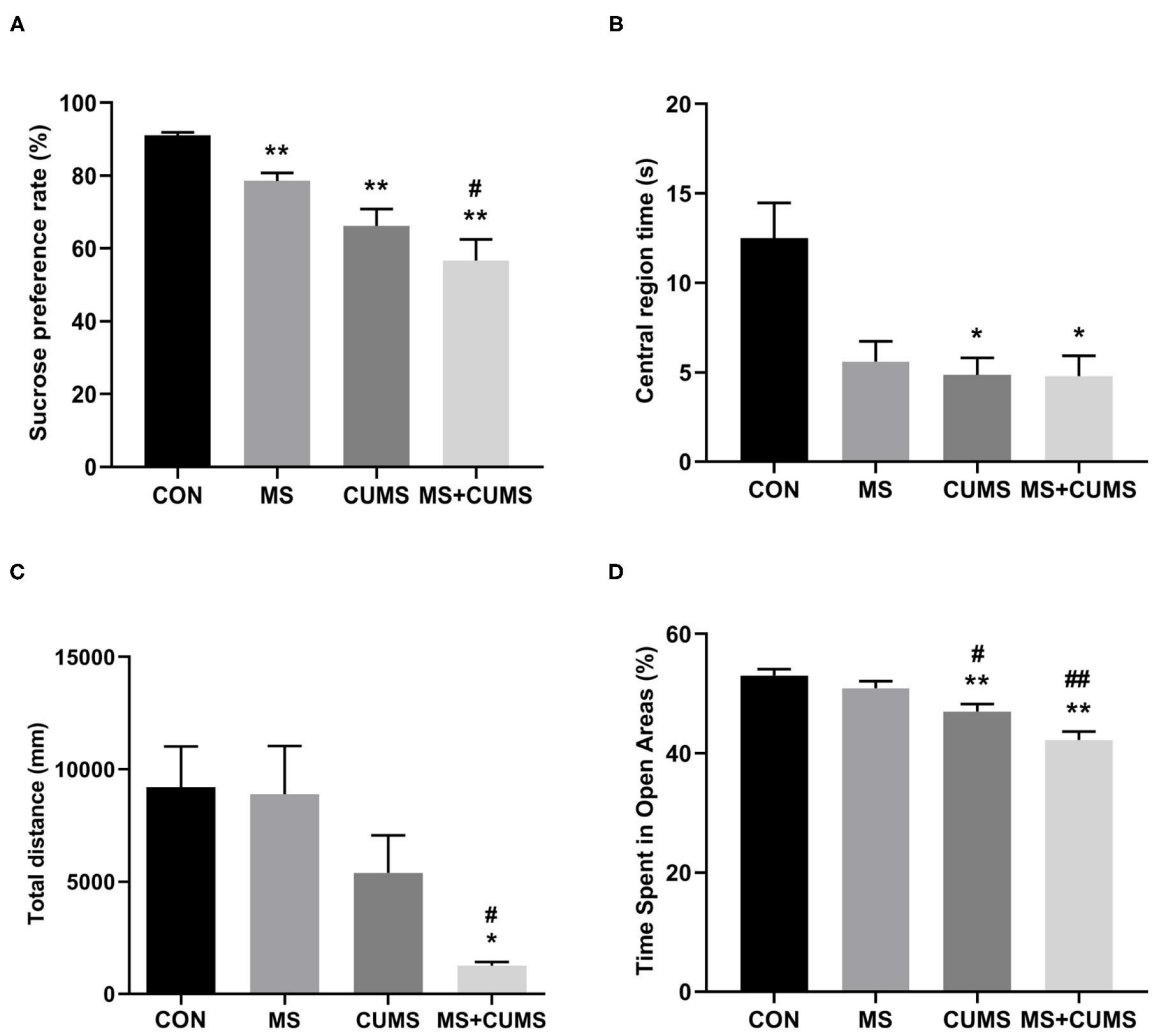
The effects of MS and CUMS on behavior in female rats. After undergoing MS and/or CUMS, all groups of rats performed behavior tests. In the sucrose preference test, the sucrose preference rate was analyzed as factors **(A)**; in the open field test, central region time **(B)** and total distance **(C)** were analyzed as factors; in the zero maze test, time spent in open areas was analyzed as factors **(D)**. The values represent the mean ± SEM, *n* = 8. ^*^p < 0.05, ^**^p < 0.01 vs. CON, ^#^*p* < 0.05, ^##^*p* < 0.01 vs. MS. CON, control; MS, maternal separation; CUMS, chronic unpredictable mild stress.

In SPT (Figure 3A), MS+CUMS group presented the lowest sucrose preference rate. MS showed a slight reduction in the sucrose preference rate, differing from the control group [*F*_(3, 28)_ = 14.64, *p* < 0.01]. The CUMS group and MS+CUMS group presented a more noticeable decline in sucrose preference rate compare with the control group [*F*_(3, 28)_ = 14.64, *p* < 0.01 and *p* < 0.01). Importantly, the sucrose preference rate reduction was the most significant in rats that underwent MS as well as CUMS, surpassing the effect of MS only [*F*_(3, 28)_ = 14.64, *p* < 0.05]. By the way, there was no difference between CUMS group and MS+CUMS group.

In OFT (Figure 3B), although the MS group, CUMS group, and MS+CUMS group showed a noticeable reduction in central region time, the MS group showed no statistical difference compared to the control group, while the CUMS group [*F*_(3, 28)_ = 6.51, *p* < 0.05] and MS+CUMS group [*F*_(3, 28)_ = 6.51, *p* < 0.05] presented a significant reduction. Double stress of MS and CUMS led to the shortest total distance among four group (Figure 3C). The MS group showed no obvious decline in total distance, and the CUMS group presented a reduction with no statistical difference. However, the total distance revealed a significant difference in MS+CUMS group compared with the control group [*F*_(3, 28)_ = 5.11, *p* < 0.05] and also with the MS group [*F*_(3, 28)_ = 5.11, *p* < 0.05].

In ZMT, MS+CUMS group showed the least time spent in open areas (Figure 3D). The CUMS group [*F*_(3, 28)_ = 13.18, *p* < 0.01] as well as MS+CUMS group [*F*_(3, 28)_ = 13.18, *p* < 0.01] presented decreased time spent in open areas compared with the control group. Notably, double stress caused the most significant reduction in time spent in open areas, even less than MS only [*F*_(3, 28)_ = 13.18, *p* < 0.01] and CUMS only [*F*_(3, 28)_ = 13.18, *p* < 0.05, not shown]. Incidentally, the CUMS group showed less time spent in open areas than the MS group [*F*_(3, 28)_ = 13.18, *p* < 0.05].

### The Effect of MS and/or CUMS on Nissl Bodies

**Figure 4:**
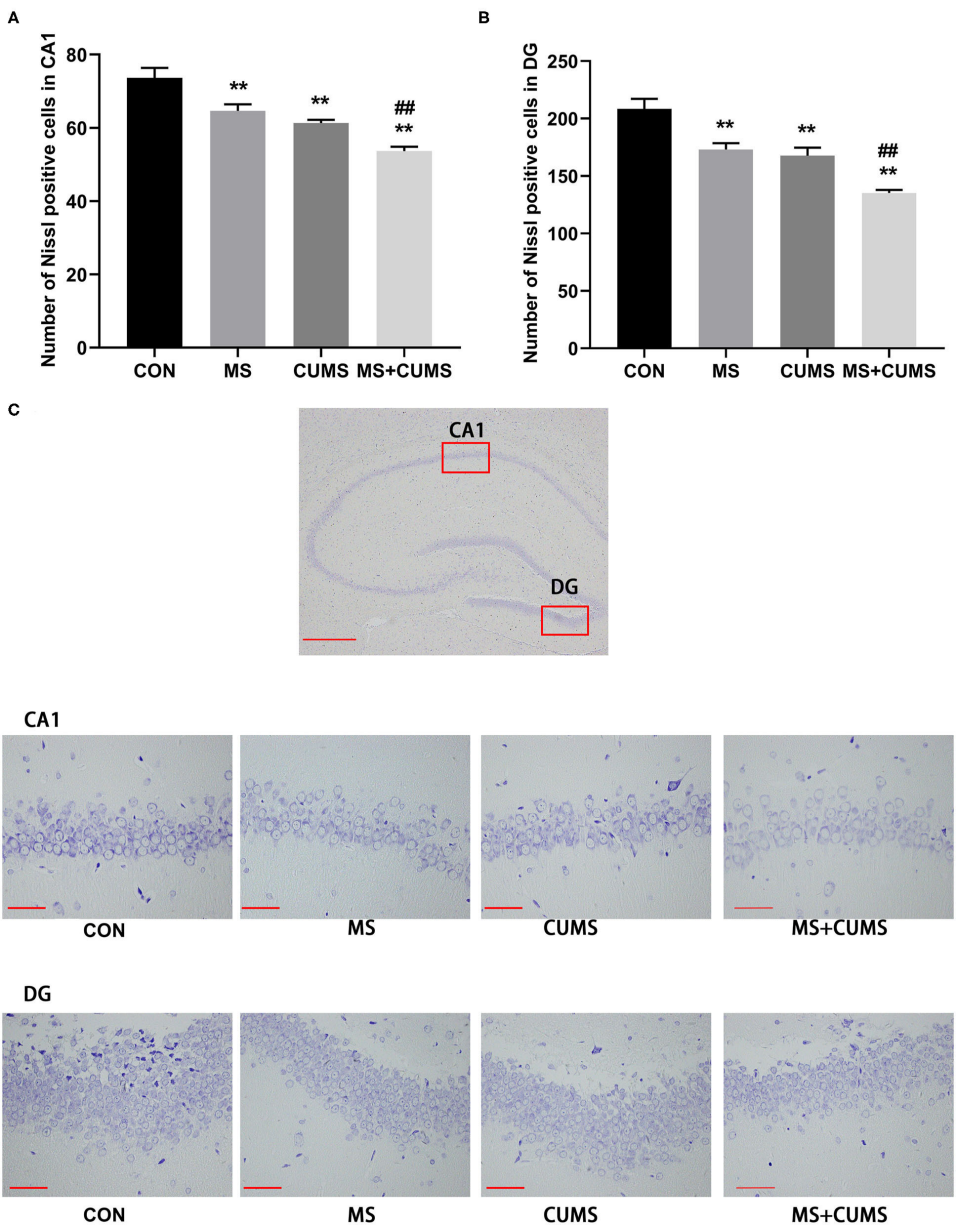
Nissl staining in the hippocampus. **(A)** Number of Nissl positive cells in CA1; **(B)** Number of Nissl positive cells in DG; **(C)** Representative photomicrograph of Nissl staining. All scale bars were 50 μm. The values represent the mean ± SEM, *n* = 3. ^**^p < 0.01 vs. CON, ^##^*p* < 0.01 vs. MS. CON, control; MS, maternal separation; CUMS, chronic unpredictable mild stress; CA1, cornu ammonis 1; DG, dentate gyrus.

According to Nissl staining, the neurons of the hippocampus in control group rats were in a large quantity and in a compact arrangement, while neurons in MS, CUMS, and MS+CUMS group rats were sparsely arranged and low in number, with neurons in the MS+CUMs group being the sparsest and the least in number (Figure 4C). Both in CA1 and DG of hippocampus, fewer Nissl positive cells were found in the MS group [CA1: *F*_(3, 8)_ = 21.50, *p* < 0.01; DG: *F*_(3, 8)_ = 21.80, *p* < 0.01] and CUMS group [CA1: *F*_(3, 8)_ = 21.50, *p* < 0.01; DG: *F*_(3, 8)_ = 21.80, *p* < 0.01) compared to the control group (Figures 4A,B). Also, both in the CA1 and DG of the hippocampus, double stress of MS and CUMS exhibited fewer Nissl-positive cells than the control group [CA1: *F*_(3, 8)_ = 21.50, *p* < 0.01; DG: *F*_(3, 8)_ = 21.80, *p* < 0.01], which is even fewer than the MS only [CA1: *F*_(3, 8)_ = 21.50, *p* < 0.01; DG: *F*_(3, 8)_ = 21.80, *p* < 0.01] and CUMS only groups [CA1: *F*_(3, 8)_ = 21.50, *p* < 0.05; DG: *F*_(3, 8)_ = 21.80, *p* < 0.01, not shown].

### The Effect of MS and/or CUMS on Expressions of Synaptic Plasticity Proteins in Hippocampus

**Figure 5:**
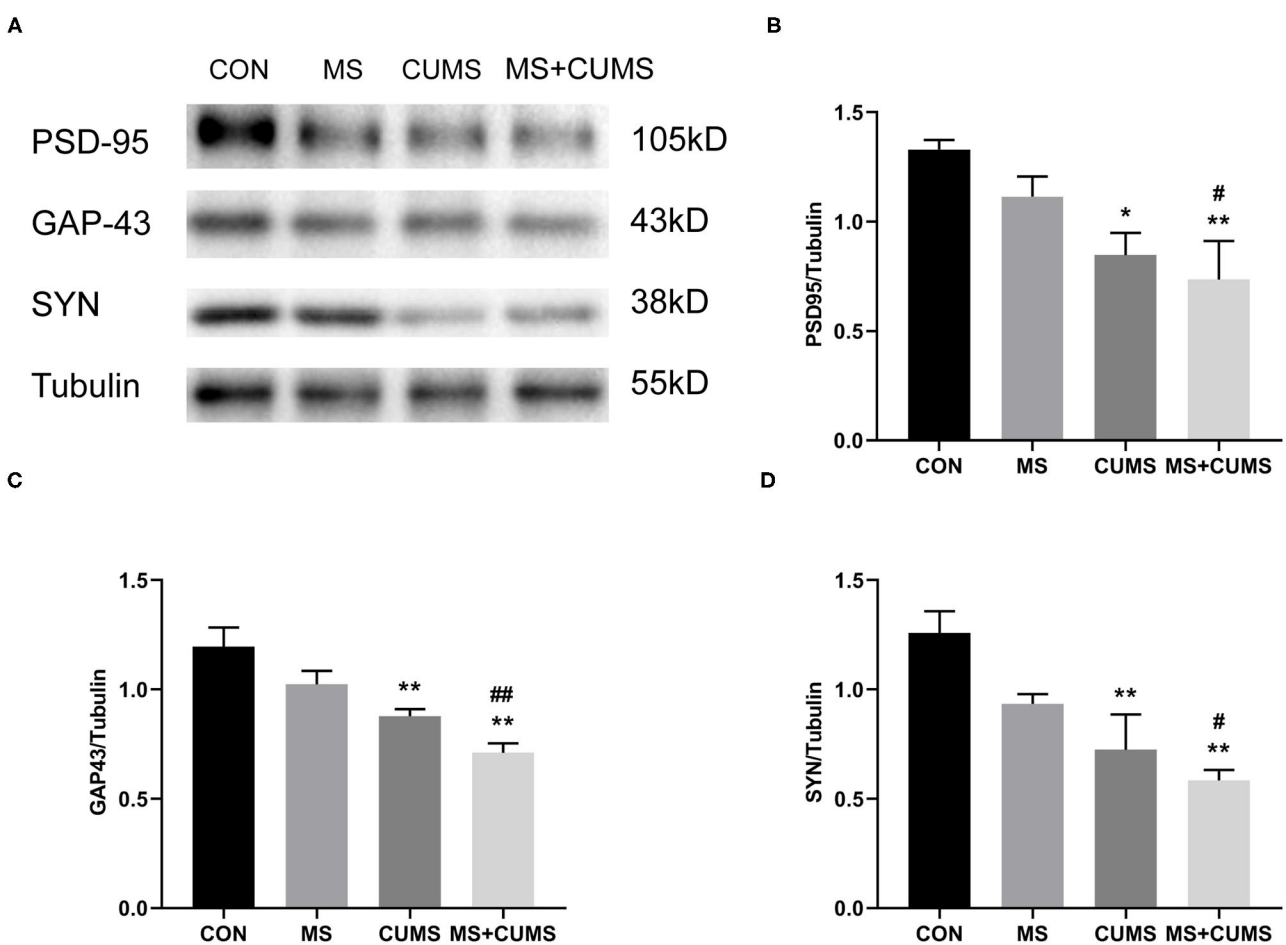
Western blot analysis. The expressions of synaptic plasticity proteins were determined by western blot **(A)**, including PSD-95 **(B)**, GAP-43 **(C)**, and SYN **(D)**. The values represent the mean ± SEM, *n* = 5. ^*^p < 0.05, ^**^p < 0.01 vs. CON, ^#^*p* < 0.05, ^##^*p* < 0.01 vs. MS. CON, control; MS, maternal separation; CUMS, chronic unpredictable mild stress.

Synaptic plasticity proteins were detected by western blot, Figure 5 shows that the expressions of PSD-95, GAP-43 and SYN in CUMS group [PSD-95: *F*_(3, 8)_ = 5.54, *p* < 0.05; GAP-43: *F*_(3, 8)_ = 11.84, *p* < 0.01; SYN: *F*_(3, 8)_ = 8.60, *p* < 0.01] and MS+CUMS group [PSD-95: *F*_(3, 8)_ = 5.54, *p* < 0.01; GAP-43: *F*_(3, 8)_ = 11.84, *p* < 0.01; SYN: *F*_(3, 8)_ = 8.60, *p* < 0.01] were downregulated. The expressions of those proteins were significantly lower in MS+CUMS group than MS group [PSD-95: *F*_(3, 8)_ = 5.54, *p* < 0.05; GAP-43: *F*_(3, 8)_ = 11.84, *p* < 0.01; SYN: *F*_(3, 8)_ = 8.60, *p* < 0.05]. Here, the double stress of MS and CUMS led to the lowest expressions of those proteins among all groups (Figure 5A).

**Figure 6:**
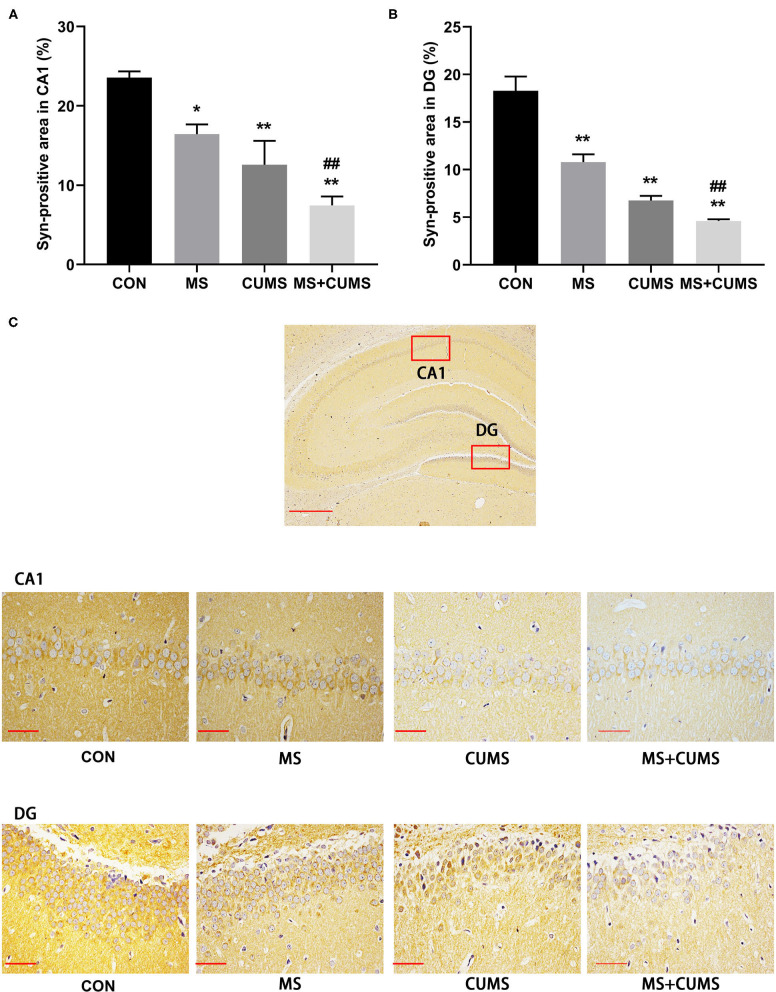
Anti-SYN immunohistochemistry analysis in the hippocampus. **(A)** Syn-positive area in CA1; **(B)** Syn-positive area in DG; **(C)** representative photomicrograph of immunohistochemistry. All scale bars were 50 μm. The values represent the mean ± SEM, *n* = 3. ^*^p < 0.05, ^**^p < 0.01 vs. CON, ^##^*p* < 0.01 vs. MS. CON, control; MS, maternal separation; CUMS, chronic unpredictable mild stress, CA1, cornu ammonis 1; DG, dentate gyrus.

In order to further determined the expression of SYN, immunohistochemistry was detected (Figure 6). Both in CA1 (Figure 6A) and DG (Figure 6B) of hippocampus, SYN expression was radically diminished in MS group [CA1: *F*_(3, 8)_ = 45.45, *p* < 0.05; DG: *F*_(3, 8)_ = 14.89, *p* < 0.01] and CUMS group [CA1: *F*_(3, 8)_ = 45.45, *p* < 0.01; DG: *F*_(3, 8)_ = 14.89, *p* < 0.01] as well as MS+CUMS group [CA1: *F*_(3, 8)_ = 45.45, *p* < 0.01; DG: *F*_(3, 8)_ = 14.89, *p* < 0.01] compared with control group. MS+CUMS group presented the least percentage of the SYN-positive area both in CA1 and DG (Figure 6C). Both in CA1 and DG of the hippocampus, double stress of MS and CUMS led to lower SYN expression than the MS group [CA1: *F*_(3, 8)_ = 45.45, *p* < 0.05; DG: *F*_(3, 8)_ = 14.89, *p* < 0.01].

## Discussion

In the present study, we examined the effects of MS, CUMS, and MS combined with CUMS on body weight, depression-like and anxiety-like behaviors, and expression of synapse-associated proteins. We found that with the experience of MS at an early age and CUMS in adolescence, adult female rats showed very intense depression-like and anxiety-like behaviors, which were more severe than those who underwent only MS or only CUMS. Moreover, depression-like and anxiety-like behaviors were related to the abnormal expressions of synapse-associated proteins, including PSD-95, GAP-43, and SYN. This study demonstrated for the first time that MS combined with CUMS induced more critical depression-like and anxiety-like behaviors than MS only or CUMS only in female rats, and the impairment of hippocampal synaptic plasticity may be the potential mechanism.

In this study, we observed the effects of MS, CUMS, and MS combined CUMS on body weight and behaviors of rats. Knowing that losing weight could be one of the symptoms of depression (27), the treatment of CUMS and MS+CUMS led to an obvious loss in weight in our study, which indicated that animals were in a progression of depression-like behavior, and this was consistent with previous studies (28, 29). Gracia-Rubio et al. reported that maternal separation caused emotional alterations but had no impact on body weight (30). Consisted with this report, our study showed that MS had no effect on body weight, while CUMS had.

Here, we revealed that rats that underwent MS showed significantly increased depression-like and anxiety-like behaviors after exposure to a CUMS environment. These findings are in agreement with previous studies showing that stress at an early stage is an important factor in the development of depression-like behaviors when exposed to adult stress, and this resulted in neurodevelopmental abnormalities through stress (31). This is consistent with studies showing that early life experience is associated with susceptibility to depression (32–35). In our study, only MS treatment of rats did not lead to anxiety-like behavior. Similarly, Chen's study showed anxiety-like behavior did not occur in MS rats (36). However, on the contrary, MS could present anxiety-like behavior in some studies (37, 38), we cannot definitely explain the different behavioral performances owing to different MS protocol and animals. So far, the opinion that a longer period of MS could induce anxiety-like behavior was confirmed in Arias' study (39). Interestingly, from the result of Susana Roque, only rats in MS_2−15_ group (MS from PND2-15) displayed depressive and anxiety-like behaviors, while rats in MS_7−20_ group (MS from PND7-20) did not display these (40). These questions need more experiment to verify.

Nissl staining is often used to detect the activity of neurons and impairment of neurons would lead to the reduction of Nissl bodies (41, 42). A small amount of Nissl positive cells was found in the hippocampus of rats underwent MS as well as CUMS. In other words, those stress may affect the activity of neurons. This conjecture is further confirmed by the following experiments.

Clinical studies have found the reduction of synaptic signaling proteins expressed in the brain regions (such as the hippocampus) with major depressive disorder patients (43, 44). SYN, PSD-95, and GAP-43 are considered to be key proteins affecting synaptic plasticity (45, 46) as well as normal signaling between neurons in the central nervous system (47). SYN is a vesicle protein of presynaptic membrane, which is closely linked to the regulation of synaptic structure and functional plasticity, which widely exists in the synaptic membrane of neurons (48) as well as rapidly recruits to the presynaptic end in response to presynaptic neuronal activity (49). In our study, low expression of SYN was detected in the hippocampus of rats that underwent MS and CUMS. Studies have shown that abnormal SYN expression is associated with depression (50). The atrophy of dendrites in the vertebral hippocampal neurons and the downregulation of SYN expression existed in chronic stress and depression (51).

PSD-95 is one of the postsynaptic densities (PSDs), which plays a key role in synaptic plasticity signal transduction (52, 53). Studies have found that most neurological diseases, such as depression, show abnormal expression of PSD-95 (54, 55). GAP-43 is located on the growth cone of the axon and participates in the synaptic plasticity of the nervous system (56, 57). In addition, GAP-43 is essential for synaptic plasticity, axon elongation, and nerve germination during the development and maturation of neurons in adult rats (58, 59). Our results are similar to Li and Yang et al. in that the expression of GAP43 in rats with depression-like and anxiety-like behaviors is significantly downregulated (60). The results showed that the expression of PSD-95 had the same trend as GAP-43. This evidence further suggested that MS combined CUMS had an effect on the impairment of synaptic plasticity. Moreover, when fewer synaptic plasticity proteins were expressed, the rats displayed more serious depression-like and anxiety-like behavior.

As the model of early MS combined with adolescence, CUMS produced critical depression-like and anxiety-like behavior as well as synaptic plasticity impairment, our finding may help to provide a novel convincing depression model. However, what is the specific mechanism? Further study would verify whether synaptic plasticity proteins be the important targets for anti-depressant interventions.

## Conclusion

In conclusion, we demonstrated that early MS combined with adolescent CUMS in female rats could induce more critical depression-like and anxiety-like behaviors. The expressions of synaptic-related proteins and the impairment of synaptic plasticity may be the potential mechanisms.

## Data Availability Statement

The datasets generated for this study are available on request to the corresponding author.

## Ethics Statement

The animal study was reviewed and approved by Animal Experimentation Committee at Guangzhou University of Chinese Medicine.

## Author Contributions

JH wrote the manuscript and conducted animal experiment. CS and RY conducted animal and analysis experiment. YS analyzed data and corrected the manuscript. WL controlled all work and revised manuscript. All authors contributed to the article and approved the submitted version.

## Conflict of Interest

The authors declare that the research was conducted in the absence of any commercial or financial relationships that could be construed as a potential conflict of interest.
